# Observation of ~100% valley-coherent excitons in monolayer MoS_2_ through giant enhancement of valley coherence time

**DOI:** 10.1038/s41377-023-01220-4

**Published:** 2023-07-13

**Authors:** Garima Gupta, Kenji Watanabe, Takashi Taniguchi, Kausik Majumdar

**Affiliations:** 1 Department of Electrical Communication Engineering, Indian Institute of Science, Bangalore, India; 2 Research Center for Functional Materials, National Institute for Materials Science, Tsukuba, Japan; 3 International Center for Materials Nanoarchitectonics, National Institute for Materials Science, Tsukuba, Japan

**Keywords:** Photonic devices, Optical properties and devices

## Abstract

In monolayer transition metal dichalcogenide semiconductors, valley coherence degrades rapidly due to a combination of fast scattering and inter-valley exchange interaction. This leads to a sub-picosecond valley coherence time, making coherent manipulation of exciton a highly challenging task. Using monolayer MoS_2_ sandwiched between top and bottom graphene, here we demonstrate fully valley-coherent excitons by observing ~100% degree of linear polarization in steady state photoluminescence. This is achieved in this unique design through a combined effect of (a) suppression in exchange interaction due to enhanced dielectric screening, (b) reduction in exciton lifetime due to a fast inter-layer transfer to graphene, and (c) operating in the motional narrowing regime. We disentangle the role of the key parameters affecting valley coherence by using a combination of calculation (solutions of Bethe-Salpeter and Maialle-Silva-Sham equations) and a careful choice of design of experiments using four different stacks with systematic variation of screening and exciton lifetime. To the best of our knowledge, this is the first report in which the excitons are found to be valley coherent in the entire lifetime in monolayer semiconductors, allowing optical readout of valley coherence possible.

## Abstract (web summary)

A demonstration of the excitons remaining fully valley coherent during their entire radiative lifetime in few-layer graphene capped monolayer MoS_2_.

## Graphical abstract

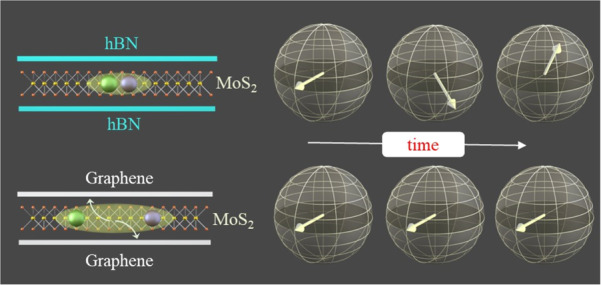

## Introduction

The bound state of an electron and a hole, an exciton, is a superposition of the conduction and valence band states in the ${\bf{K}}$ and ${{\bf{K}}}^{{\boldsymbol{{\prime} }}}$ valleys in monolayer transition metal dichalcogenides (TMDs)^1–3^. ${\bf{K}}$ and ${{\bf{K}}}^{{\boldsymbol{{\prime} }}}$ valley excitons are selectively generated by circularly polarized light excitation of opposite helicities^4–7^. On linearly polarized excitation, a hybrid ${\bf{K}}-{{\bf{K}}}^{{\boldsymbol{{\prime} }}}$ exciton is generated in a state of valley coherence^8,9^. However, valley coherence degrades rapidly due to a combined effect of fast scattering and inter-valley exchange^10–12^. The reported values of valley coherence time lie in the range of $98-520$ fs^12–17^, much shorter than the exciton radiative lifetime of $\sim 1$ ps^18–20^. This makes optical read out of strong exciton valley coherence a highly challenging task. To be able to use these coherent excitons as a qubit for quantum information processing, a longer valley coherence time is desirable to perform any manipulation on it. Any technique^21^ that enhances this valley coherence time significantly is thus of high scientific importance.

Here we demonstrate a $100 \%$ degree of linear polarization (DOLP) in photoluminescence (PL) peak of ${A}_{1s}$ exciton in a monolayer of MoS_2_ encapsulated with few-layer-graphene (FLG) at the top and bottom. Such a complete retention of the generated valley coherence in steady-state PL implies the achievement of a large valley coherence time, the measured value of which is only limited by the lifetime of the exciton. This suggests that the valley coherence time has been significantly enhanced as compared to the reported values to date^12–16^.

## Results

**Fig. 1:**
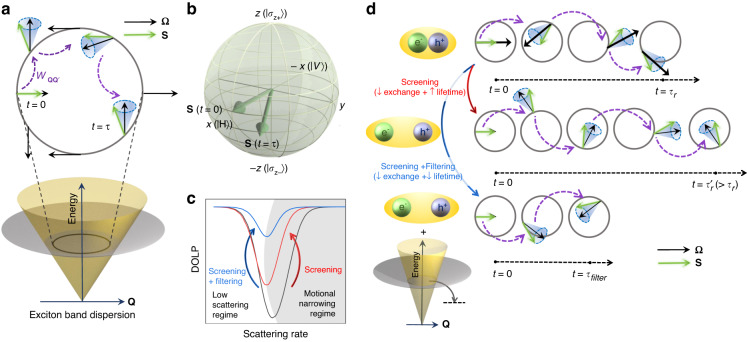
Mechanism of exciton valley decoherence and the factors affecting it. **a** Top view of a ring inside the light cone of the exciton band showing the exciton decoherence dynamics due to scattering ${{\rm{W}}}_{{\bf{Q}}{{\bf{Q}}}^{{\boldsymbol{{\prime} }}}}$ within the light cone (purple dashed arrows) and subsequent precession because of inter-valley exchange induced pseudo-magnetic field (black solid arrows). **b** The direction of **S** at generation $(t=0)$ and recombination $(t=\tau )$ are shown in the Bloch sphere. **c** The calculated DOLP as a function of scattering rate with motional narrowing regime shown in shade. **d** The decoherence of **S** (green arrows) with time due to scattering (dashed purple arrow) and precession around **Ω** (black arrows) for an exciton for three different scenarios. The effect of screening versus screening + filtering on valley decoherence is compared in the three rows

Depending on the linear polarization direction of the excitation light, the excitons are generated at specific center-of-mass momentum $({\bf{Q}}{\boldsymbol{)}}$ values [where ${\bf{Q}}={{\bf{k}}}_{{\bf{e}}}{\boldsymbol{+}}{{\bf{k}}}_{{\bf{h}}}$, ${{\bf{k}}}_{{\bf{e}}}{\boldsymbol{(}}{{\bf{k}}}_{{\bf{h}}}{\boldsymbol{)}}$ denoting the electron (hole) crystal momentum] in the exciton band at time $t=0$ (Fig. 1). During its lifetime, the exciton undergoes scattering and exchange interaction, which, coupled together, degrades the valley coherence. The polarization state can be represented by the pseudospin vector ${\bf{S}}$ in the Bloch sphere. At $t=0,$ the direction of ${\bf{S}}$ is parallel to the exchange-induced magnetic field (denoted by the precession frequency ${\bf{\Omega }}$). Considering $x-$ polarized excitation, the system is generated in a pure state represented by ${S}_{x}=1$, ${S}_{y}=0,\,{S}_{z}=0$. When excitons scatter to different ${\bf{Q}}$ values, the pseudospin precesses on experiencing a finite torque around ${\bf{\Omega }}{\boldsymbol{(}}{\bf{Q}}{\boldsymbol{)}}$ due to which it becomes a mixed state represented by a density matrix operator $\rho$. The ${\bf{Q}}$-space and the Bloch sphere representations of this mechanism are shown in Fig. 1a, b. On decoupling the density matrix in terms of the number trace and traceless matrix ${\bf{S}}.{\boldsymbol{\sigma }}$ (where ${\boldsymbol{\sigma }}$ denotes the Pauli matrices), the overall dynamics of the valley pseudospin as described by the Maialle-Silva-Sham (MSS) mechanism (see Supplementary Note 1 for proof) is given by^10^:

1 $$\frac{d{\bf{S}}({\bf{Q}})}{{dt}}={\bf{\Omega }}\left({\bf{Q}}\right)\times {\bf{S}}\left({\bf{Q}}\right)+\sum _{{{\bf{Q}}}^{{\prime} }}{{\rm{W}}}_{{\bf{Q}}{{\bf{Q}}}^{{\prime} }}\left[{\bf{S}}\left({{\bf{Q}}}^{{\prime} }\right)-{\bf{S}}\left({\bf{Q}}\right)\right]-\frac{1}{\tau }{\bf{S}}\left({\bf{Q}}\right)+{\bf{G}}$$

Here ${{\rm{W}}}_{{\bf{Q}}{{\bf{Q}}}^{{\boldsymbol{{\prime} }}}}$ is the rate of any generic momentum scattering mechanism, e.g., exciton-impurity and exciton-phonon scattering. $\tau$ is the net exciton lifetime given by $1/\tau \,=\,1/{\tau }_{r}+1/{\tau }_{{nr}}+\,1/{\tau }_{{filter}}$, where ${\tau }_{r},\,{\tau }_{{nr}},$ and ${\tau }_{{filter}}$ are the radiative, non-radiative, and filtering timescale. Filtering is a non-radiative process where the excitons are scattered out of the light cone, e.g., scattering to lower energy states, interlayer transfer to graphene, etc., in which case, the light collection is limited to $t\le {\tau }_{{filter}}$. ${\bf{G}}$ represents the exciton generation rate. On recombination, the DOLP of this mixed state is given by $\left\langle {S}_{x}\right\rangle$, averaged over ${\bf{Q}}$ values within the light cone (see Supplementary Note 2 for proof).

The possible ways to improve the valley coherence time are: (a) by minimizing scattering $({{\rm{W}}}_{{\bf{Q}}{{\bf{Q}}}^{{\boldsymbol{{\prime} }}}})$ inside the light cone such that ${\bf{S}}$ does not accumulate random phase by precessing around ${\bf{\Omega }}\left({\bf{Q}}\right)$, or by enhancing ${{\rm{W}}}_{{\bf{Q}}{{\bf{Q}}}^{{\boldsymbol{{\prime} }}}}$ such that the whole operation is pushed towards the motional narrowing regime (simulation results in Fig. 1c); (b) by screening the electron–hole interaction which results in reduced exchange interaction, and in turn a suppressed ${\bf{\Omega }}$ (middle panel of Fig. 1d). However, a side-effect of the enhanced screening is an increment in the exciton lifetime due to a reduction in the binding energy. This can be overcome by (c) reducing $\tau$ by introducing a fast-filtering mechanism^21–23^ (bottom panel of Fig. 1d).

**Fig. 2:**
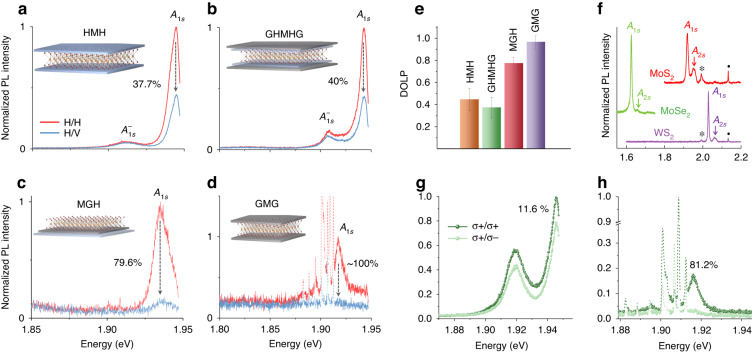
Excitonic emission from different stacks along with DOLP and DOCP. **a**–**d** PL spectra with near-resonant 633 nm linearly polarized excitation in co-(H/H) and cross- (H/V) polarized detection configuration in the **a** hBN-MoS_2_-hBN (HMH) stack, **b** FLG-hBN-MoS_2_-hBN-FLG (GHMHG) stack, **c** MoS_2_-FLG-hBN (MGH) stack, and the **d** FLG-MoS_2_-FLG (GMG) stack at *T* = 5 K. ${A}_{1s}$ and ${A}_{1s}^{-}$ represent the exciton and the trion peak, respectively. **e** Bar graph comparing the experimental DOLP values along with the error bars in the four stacks. **f** PL spectra obtained from FLG-TMD-FLG stack (using 532 nm excitation) for monolayer MoS_2_, MoSe_2_, and WS_2_ showing the prominent ${A}_{1s}$ and ${A}_{2s}$ peaks. The ${A}_{2s}-{A}_{1s}$ separation is around $44\,(32)$ meV in MoS_2_ (MoSe_2_, WS_2_). The peaks marked as * and **⋅** are the 2D and the G Raman peaks of the FLG. **g**, **h** Representative PL spectra taken with circularly polarized excitation in co-$\,(\sigma +/\sigma +)$ and cross- $(\sigma +/\sigma -)$ polarized detection configuration. The corresponding DOCP value of the ${A}_{1s}$ exciton in (**g**) the HMH and (**h**) the GMG stack at *T* = 5K is shown in the inset. The peaks indicated by the dashed lines in (**d**), (**h**) represent the prominent Raman peaks in the GMG stack due to dual resonance

To understand the interplay among these factors systematically, we prepare four different stacks of monolayer MoS_2_ combined with hBN and FLG, which are: (1) hBN-MoS_2_-hBN (HMH), (2) FLG-hBN-MoS_2_-hBN-FLG (GHMHG), (3) MoS_2_-FLG-hBN (MGH), and (4) FLG-MoS_2_-FLG (GMG) (see Methods). We obtain an exciton DOLP of $44.5\,(\pm 10) \%$ in the HMH stack, $37\,(\pm 9) \%$ in the GHMHG stack, $77\,(\pm 5) \%$ in the MGH stack, and $96\,(\pm 6) \%$ in the GMG stack on 633 nm near resonant laser excitation at 5 K. Linear polarization-resolved representative PL spectra in Fig. 2a–d and the bar diagram (with error bars) in Fig. 2e compare the DOLP numbers in all the four stacks (more spectra in Figs. S3-S6). Interestingly, there are several spots where we observe $\sim 100 \%$ DOLP in the GMG stack (Fig. 2d and Fig. S6). In Fig. S7a, we show similar results of ~100% DOLP obtained from few-layer graphene encapsulated monolayer WS_2_ (GWG) stack. We also perform polarization dependent time-resolved photoluminescence (TRPL) measurement and obtain a peak DOLP of 97.6% in the GWG stack (see Fig. S7b).

We would like to highlight some additional observations on the GMG stack before the main analysis begins: (1) As a result of FLG encapsulation, the PL spectra predominantly consist of the clean ${A}_{1s}$ exciton peak^22^. The spectra of FLG encapsulated monolayer MoS_2_, MoSe_2_, and WS_2_ on 532 nm excitation are shown in Fig. 2f, clearly indicating suppression of spurious peaks from defect-bound excitons and other excitonic complexes. We also observe a clear ${A}_{2s}$ peak located at 44 (32) meV higher than the ${A}_{1s}$ peak in MoS_2_ (MoSe_2_ and WS_2_) due to enhanced screening^24^. (2) We also get a very high degree of circular polarization (DOCP) of $81.6\,(\pm 2)\, \%$ in the GMG stack, much larger compared to the $20.5\,(\pm 9) \%$ DOCP in the HMH stack (Fig. 2g, h and more spectra in Fig. S8-S9). The in-plane nature of ${\bf{\Omega }}$ explains this observation that DOCP is smaller than DOLP for 2D excitons, which is consistent with previous reports^10,25^ (see Supplementary Note 3). This indicates that starting with a linear polarization (that is, on the equator of the Bloch sphere) is the most favourable scenario to maintain valley coherence compared with any other (elliptical) polarization. (3) The initial and the final state in the Raman scattering process coincides with the ${A}_{2s}$ and the ${A}_{1s}$ exciton level, respectively, on 633 nm excitation at 5 K in the GMG stack. This dual resonance enhances the intensity of the Raman peaks significantly (represented by the dashed lines in Fig. 2d, h) and enables the observation of other less commonly observed modes distinctly (Fig. S10). The fact that the excitation laser is resonant with the 2s state in the GMG stack and we observe almost fully coherent 1s excitonic emission from the stack, it is likely that the generated 2s excitons relax through polarization preserving processes, such as, dipole-coupled radiative transition (2s → 2p → 1s).

**Fig. 3:**
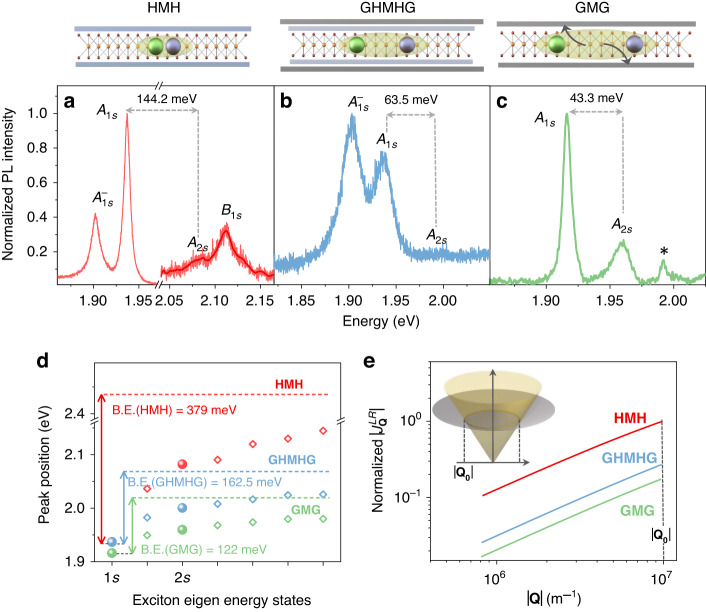
Evidence of graphene induced screening of electron-hole exchange interaction. **a**–**c** Top panel: Schematic representation of the screening effect in the different stacks. Bottom panel: PL spectra taken with 532 nm excitation highlighting the different degree of dielectric screening in our samples. The ${A}_{2s}-{A}_{1s}$ separation for the HMH stack (144.5 meV), GHMHG stack (63.5 meV), and the GMG stack (43.3 meV) is indicated by the dashed arrows. * in (**c**) is the 2D Raman peak of FLG. **d** Eigen energies (open symbols) and the corresponding binding energies of the ${A}_{1s}$ exciton obtained from the Bethe-Salpeter equation. The solid symbols denote experimental data. The dashed lines are the corresponding calculated continuum levels. **e** Calculated value of the normalized long-range exchange potential variation inside the light cone $(\left|{\bf{Q}}\right| < \left|{{\bf{Q}}}_{{\bf{0}}}\right|)$. Inset: light emitting region $(\left|{\bf{Q}}\right| < \left|{{\bf{Q}}}_{{\bf{0}}}\right|)$ of the exciton band highlighted by the light cone

In order to establish the different degrees of screening in the stacks, we plot the PL spectra for the HMH, GHMHG, and the GMG stack obtained from 532 nm excitation in Fig. 3a–c. The ${A}_{2s}-{A}_{1s}$ energy separation obtained is 144.5 meV in the HMH stack, which reduces to 60 and 45 meV in the GHMHG and GMG stack, respectively. To get an estimate of the ${A}_{1s}$ exciton binding energy change (Fig. 3d), we obtain the continuum of the exciton energy spectrum by numerically solving the Bethe-Salpeter equation^26^ using a two-band Hamiltonian. In the calculation, the parameters are fitted such that the experimentally obtained ${A}_{2s}-{A}_{1s}$ energy separation matches with the calculated one (Supplementary Note 4 and Fig. S2). The calculated ${A}_{1s}$ exciton binding energy is 379 meV in the HMH system, which reduces to 162.5 and 122 meV in the GHMHG and the GMG stacks respectively due to graphene induced screening.

One immediate consequence of such a screening is the reduction in the inter-valley exchange interaction^25^. The exchange interaction is composed of two components—the short-range part and the long-range part. The short-range component is zero at **Q** **=** **0** due to the three-fold rotational symmetry condition and is negligible at higher **Q** values. The long-range part is given by^27^:

2 $$\,{J}_{{\bf{Q}}}^{{LR}}\propto -\frac{{\left|\sum _{{\bf{k}}}\psi \left({\bf{k}}\right)\right|}^{2}}{{E}_{g}^{2}}V\left({\bf{Q}}\right){\left|{\bf{Q}}\right|}^{2}$$

Here $\frac{{\left|\sum _{{\bf{k}}}\psi ({\bf{k}})\right|}^{2}}{A}\,=\,{\left|\psi ({r}_{{eh}}=0)\right|}^{2}$ is the electron–hole wavefunction overlap at zero relative separation $\left({r}_{{eh}}=0\right)$, **k** is the reciprocal space wave-vector, ${E}_{{g}}$ denotes the bandgap of MoS_2_, and $V({\bf{Q}})$ is the electron–hole coulomb interaction. The dielectric screening modulates the following factors: (a) ${\left|\psi ({r}_{{eh}}=0)\right|}^{2}$ - due to a reduction in the 2D exciton binding energy^24,28^; (b) ${E}_{g}$ - due to bandgap renormalization effect in monolayer TMDs^29,30^; and (c) $V({\bf{Q}})$ - due to suppressed electron-hole interaction^31^. In Fig. 3e, we show the variation in ${J}_{{\bf{Q}}}^{{LR}}$ with **Q** within the light cone, and hence the screening induced suppression of the long-range exchange in our samples (see Supplementary Note 4).

Another consequence of screening is the enhancement of the exciton radiative lifetime due to a reduced electron–hole wavefunction overlap. Here, a longer lifetime is undesirable as it leads to a larger valley decoherence (Fig. 1a). To estimate the exciton lifetime and its role in the valley decoherence, we carry out TRPL measurements on our samples (see Methods and Fig. S11). The TRPL values obtained in our stacks are as follows: <5 ps in the HMH stack, 6–8 ps in the GHMHG stack, and <5 ps in the GMG stack. The uncertainty in the lifetime in the HMH and GMG stacks arises as it is smaller than the 10% limit of our Instrument Response Function (IRF) width^32^. Nonetheless, several reports supporting these numbers are already available in literature^18,19,22,33^. Moreover, the qualitative trend in the exciton lifetime testifies the anticipated trend in Fig. 1d, showing a clear enhancement in the exciton lifetime in the GHMHG stack, as compared to the other two stacks.

Obtaining an average DOLP of only 44.5 (±10)% implies an ultra-short valley coherence time in the HMH stack, in agreement with previous reports^12,15^. On the other hand, in the GHMHG stack, an increased exciton lifetime is an evidence of screening induced enhancement of exciton lifetime as a result of introducing top and bottom FLG. Due to the opposing roles of reduced exchange and increased lifetime, we do not observe any improvement in the exciton DOLP in this sample compared with the HMH sample.

This side-effect of screening driven enhanced exciton lifetime is eliminated in the GMG stack through filtering, where light collection from the long-lived excitons is prohibited due to a fast transfer of excitons to graphene. The extracted timescale corresponds to the graphene-transfer-limited exciton lifetime in this system. This timescale is similar to that in the HMH stack, and in agreement with previous report^22,33^. Therefore, the significant exciton DOLP difference between the HMH and the GMG stacks is attributed to screening modified exchange interaction without any confounding effect due to a change in the exciton lifetime.

To obtain a quantitative understanding, we solve the steady-state form of the MSS equation:

3 $${\bf{G}}=\frac{1}{\tau }{\bf{S}}\left({\bf{Q}}\right)-{\bf{\Omega }}\left({\bf{Q}}\right)\times {\bf{S}}\left({\bf{Q}}\right)-\sum _{{\bf{Q}}^{\boldsymbol{\prime}}}\frac{w}{{Q}^{2}\,{\sin }^{2}\frac{\alpha }{2}}\left[{\bf{S}}\left({{\bf{Q}}}^{{\boldsymbol{{\prime} }}}\right)-{\bf{S}}\left({\bf{Q}}\right)\right]$$

**Fig. 4:**
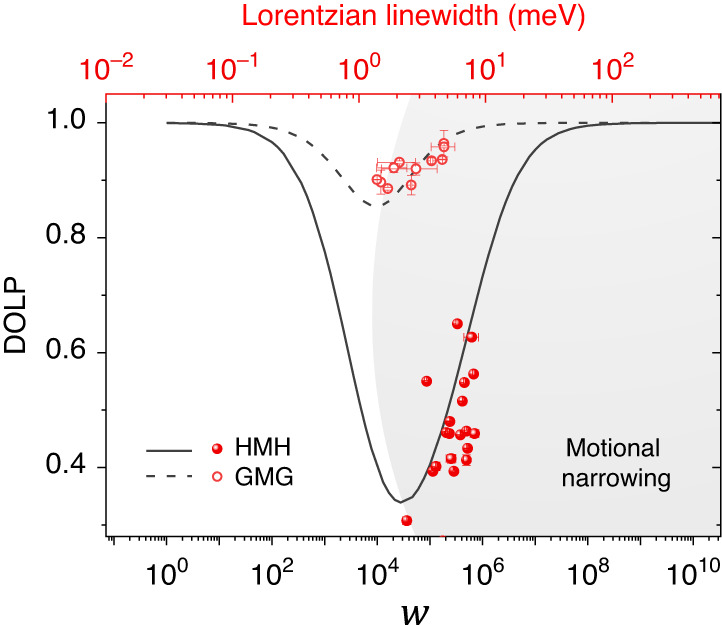
Comparison of the experimental data with the steady-state solution of the MSS equation. Simulation results comparing the exciton DOLP $\left(\left\langle {S}_{x}\right\rangle \right)$ as a function of the scaling factor $\left(w\right)$ of the scattering rate (bottom axis) for the HMH stack (solid black trace) and the GMG stack (dashed black trace). The downward trend in the left-hand side (unshaded region) is the low-scattering regime, and the upward trend in the right-hand side (shaded region) is the motional narrowing regime. Overlapped on the simulation results is the experimentally obtained DOLP variation with the Lorentzian linewidth $\left({\Gamma }_{\hom }\right)$ of the corresponding co-polarized PL spectrum (top axis) for the HMH stack (solid spheres) and the GMG stack (open circles). The upward trend of the experimental data suggests that both the stacks are operating in the motional narrowing regime

We obtain the DOLP $(\left\langle {S}_{x}\right\rangle )$ for the ${A}_{1s}$ exciton in the HMH and the GMG stack (Supplementary Note 4). For ${{\rm{W}}}_{{\bf{Q}}{{\bf{Q}}}^{{\boldsymbol{{\prime} }}}}$, the exciton-impurity scattering rate expression is used (Supplementary Note 4.2). $\tau$ is the exciton lifetime, $\alpha$ is the angle between the initial (**Q**) and the final $\left({{\bf{Q}}}^{{\boldsymbol{{\prime} }}}\right)$ state and $w$ is an overall scaling factor. We neglect the contribution of exciton-phonon scattering in decoherence at 5 K. Plotte d in Fig. 4 is the variation in $\left\langle {S}_{x}\right\rangle$ as a function of the $w$ for the two stacks. In both the cases, the V shaped variation is understood as follows: For small $w$, an increase in the scattering degrades the valley coherence due to enhanced exciton precession around **Ω** (Fig. 1a). However, this effect is non-monotonic, as on significantly enhancing the scattering rate, the DOLP starts increasing after reaching a minimum. This phenomenon is referred to as motional narrowing^10,34^, and arises due to a cancellation of the accumulated randomness in the phase information of **S**. Mathematically, the system is in the motional narrowing regime when the exciton scattering frequency becomes larger than the precession frequency, leading to a longer pseudospin coherence time.

We take the extracted homogeneous (Lorentzian component of the Voigt fitting) linewidth (${\Gamma }_{\hom }$) of the co-polarized exciton PL peak as the experimental analogue of the scattering rate. The experimental DOLP as a function of the Lorentzian linewidth, superimposed on the simulation results, is shown in Fig. 4. Both in GMG and HMH stacks, the extracted value of ${\Gamma }_{\hom }$ is much larger than the exciton lifetime limited linewidth, as obtained from TRPL. This suggests that the impurity scattering rate is similar in both the samples, and it dominates over other linewidth broadening mechanisms. This is also evident from the upward trend of the experimental DOLP with ${\Gamma }_{\hom }$, which is in excellent agreement with the rising side of $\left\langle {S}_{x}\right\rangle$ versus $w$ in the simulation. This suggests that the whole operation lies in the motional narrowing regime in both the samples.

## Discussion

We conclude that the combined effect of enhanced screening, reduced lifetime due to interlayer transfer, and motional narrowing helps us to achieve ~100% exciton DOLP in our FLG-capped MoS_2_ sample. This is a direct evidence of valley coherence being maintained during the entire exciton lifetime by cutting down the decoherence channels before the spontaneous emission. Achieving ~100% steady-state DOLP also indicates that the true valley coherence timescale is beyond few picoseconds in the GMG stack, which is in excellent agreement with simulation result obtained by solving time-dependent MSS equation (see Fig. S12).

The combination of such ~100% polarization, coupled with background-free, narrow linewidth emission, makes the GMG stack a promising substrate for spectral diffusion-free, indistinguishable single photon source. As the initialized coherence in the exciton is shown to be staying protected for a longer time, the results have intriguing prospects on performing experiments involving coherent manipulation of exciton and building quantum system operating at these timescales.

## Methods

### Sample preparation

All the stacks in this paper are prepared first by mechanically exfoliating the layered material on a Polydimethylsiloxane (PDMS) film, and then its subsequent transfer in a controlled manner underneath a microscope on a Si substrate covered with 285 nm thick thermally grown SiO_2_. The thickness of the few-layer graphene is chosen to be 2–3 nm in all the stacks. The thickness of hBN layers is in the range of 20–30 nm apart from the GHMHG stack. To ensure strong screening in the GHMHG stack, the hBN thickness is kept at ~5 nm. After the preparation of the entire stack, the samples are annealed at 200 °C for 5 h (pressure ~10^-6^ torr) to ensure better adhesion between successive layers.

### Sample characterization

All the measurements are taken in a closed-cycle optical cryostat (Montana Instruments) at 5 K using a ×50 long-working-distance objective having a numerical aperture of 0.5. To measure the exciton DOLP $\left[=\,({I}_{H/H}-{I}_{{\rm{H}}/{\rm{V}}})/\left({I}_{H/H}+{I}_{{\rm{H}}/{\rm{V}}}\right)\right]$, we place the analyzer in the parallel $\left({I}_{H/H}\right)$ and perpendicular $\left({I}_{{\rm{H}}/{\rm{V}}}\right)$ direction in the collection path relative to the excitation polarization direction. For the DOCP measurements, a quarter-wave plate is inserted just before the objective lens, and aligned at 45° with respect to the incoming linearly polarized light. The time-resolved photoluminescence measurement is carried out using a 531 nm laser controlled by the PDL-800 D driver (laser pulse width is 48 ps). We use a single photon counting detector from Micro Photon Devices and the time correlated measurements are taken using the PicoHarp 300 TCSPC system (PicoQuant). We use a combination of two bandpass filters to get the time resolved counts of the ${A}_{1s}$ exciton at 5 K: 650 (FWHM - 55 nm) and 635 nm bandpass filter (FWHM - 10 nm) for the HMH and the GHMHG stack, and 650 (FWHM - 55 nm) and 660 nm bandpass filter (FWHM - 10 nm) for the GMG stack. The instrument response function (IRF) has an FWHM of 52 ps, and shows a decay of 23 ps. The deconvolution of the TRPL data with the IRF is carried out using the QuCoa software (PicoQuant).

## Supplementary information

Supplementary Information
