# Supplementary material for: Observation of ~100% valley-coherent excitons in monolayer MoS2 through giant enhancement of valley coherence time
Source: Light Sci Appl. 2023 Jul 13;12:173. doi: 10.1038/s41377-023-01220-4 (PMC10344883; doi:10.1038/s41377-023-01220-4)
Supplement: Supplementary file 1 — Supplementary Information [file 41377_2023_1220_MOESM1_ESM.pdf]

Supplementary Information for Observation of ~100% valley-coherent excitons in monolayer MoS_2 through giant enhancement of valley coherence time

Garima Gupta[1], Kenji Watanabe[2], Takashi Taniguchi[3], and Kausik Majumdar[1,*]

[1]*Department of Electrical Communication Engineering, Indian Institute of Science, Bangalore 560012, India*

[2] *Research Center for Functional Materials, National Institute for Materials Science, 1-1 Namiki, Tsukuba 305-044, Japan*

[3] *International Center for Materials Nanoarchitectonics, National Institute for Materials Science, 1-1 Namiki, Tsukuba 305-044, Japan*

**corresponding author, E-mail: kausikm@iisc.ac.in*

Supplementary Note 1: Derivation of the Maialle-Silva-Sham (MSS) equation

Consider an exciton pseudospin **S** pointing in the (θ, ϕ) direction on the Bloch sphere as shown below.

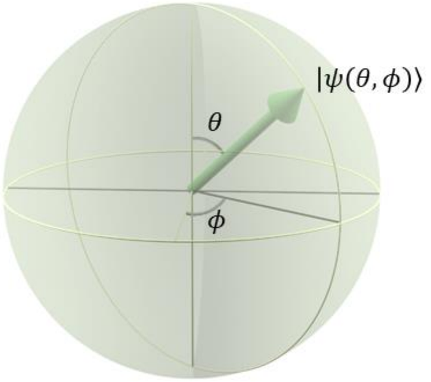

Figure S1: A schematic representation of the Bloch sphere indicating the exciton pseudospin direction.

This exciton state can be written in the basis of polar states $|\sigma_{z+}\rangle$ and $|\sigma_{z-}\rangle$ as:

$$|\psi(\theta,\phi)\rangle = \begin{pmatrix} \cos\frac{\theta}{2} \\ e^{i\phi}\sin\frac{\theta}{2} \end{pmatrix}$$

The density matrix form of this pure state single qubit system is given by:

$$\rho(\theta,\phi) = |\psi(\theta,\phi)\rangle\langle\psi(\theta,\phi)|$$

which may be written in terms of the identity matrix I and Pauli Matrices as:

$$\rho(\theta,\phi) = \frac{1}{2}\left(\mathrm{I} + \sin\theta\cos\phi\ \sigma_x + \sin\theta\sin\phi\ \sigma_y + \cos\theta\ \sigma_z\right)$$

We denote the projection of **S** along the x, y and z axis of the Bloch sphere as $S_x = \sin\theta\cos\phi$, $S_y = \sin\theta\sin\phi$ and $S_z = \cos\theta$. S_x, S_y and S_z are the coefficients representing the expectation value of angular momentum along that direction. In terms of polarization, the x, y axes on the equator of the Bloch sphere correspond to the linearly polarized light along x axis and 45^0 from x axis, and the polar points correspond to the circularly polarized light in the real space.

The time evolution of the density operator ρ for the mixed state in the presence of exchange field and momentum scattering $\left(W_{\mathbf{QQ}'}\right)$ *is* given by MSS as[1]:

$$\frac{d\rho(\mathbf{Q},t)}{dt} = \frac{i}{\hbar}[\rho(\mathbf{Q},t),H] + \sum_{\mathbf{Q}'} W_{\mathbf{QQ}'}[\rho(\mathbf{Q}',t) - \rho(\mathbf{Q},t)] - \frac{\rho(\mathbf{Q},t)}{\tau} + G \qquad (1)$$

Here τ represents the net exciton lifetime within the light cone, and the matrix G accounts for the exciton generation rate. H is the total pseudospin Hamiltonian which can be decomposed into $H = H_0 + H_1(\mathbf{Q})$, where H_0 is the diagonal matrix that accounts for the applied magnetic field in the out-of-plane direction:

$$H_0 = \begin{bmatrix} E_+ & 0 \\ 0 & E_- \end{bmatrix}$$

and H_1 is the matrix containing the off-diagonal elements that is responsible for the exchange driven exciton pseudospin flip:

$$H_1(\mathbf{Q}) = \frac{\hbar\Omega_{\parallel}(\mathbf{Q})}{2}\begin{bmatrix} 0 & e^{-i2\phi} \\ e^{i2\phi} & 0 \end{bmatrix}$$

where ϕ is the angle between $\mathbf{Q}$ and the x axis.

H_0 can be expanded as

$$H_0 = \frac{1}{2}Tr(H_0)\mathrm{I} + \frac{\hbar\Omega_0}{2}\sigma_z$$

where $\Omega_0 = (E_+ - E_-)/\hbar$, and $H_1(\mathbf{Q})$ can be written as

$$H_1(\mathbf{Q}) = \frac{\hbar\,\Omega_{\parallel}(\mathbf{Q})}{2}\left[\cos(2\phi)\,\sigma_x + \sin(2\phi)\,\sigma_y\right].$$

Thus, we obtain

$$H = \frac{1}{2}Tr(H_0)\mathrm{I} + \frac{\hbar}{2}\boldsymbol{\Omega}.\boldsymbol{\sigma}$$

where $\boldsymbol{\Omega} = [\Omega_{\parallel}\cos\phi,\ \Omega_{\parallel}\sin\phi,\ \Omega_0]$. Similarly, decomposing the density matrix in terms of its trace N and the traceless part $\mathbf{S}.\boldsymbol{\sigma}$, where $\boldsymbol{\sigma}$ denotes the Pauli matrices, the commutator $[\rho(\mathbf{Q},t),H\,]$ turns out to be:

$$[\rho(\mathbf{Q},t),H] = \frac{\hbar}{2}[\mathbf{S}(\mathbf{Q},t).\boldsymbol{\sigma},\boldsymbol{\Omega}.\boldsymbol{\sigma}] \qquad (2)$$

Combining equations (1) and (2):

$$\frac{d}{dt}\left(\frac{N(\mathbf{Q},t)}{2}\mathrm{I} + \mathbf{S}(\mathbf{Q},t).\boldsymbol{\sigma}\right) = \frac{i}{2}[\mathbf{S}(\mathbf{Q},t).\boldsymbol{\sigma}, \boldsymbol{\Omega}.\boldsymbol{\sigma}]$$

$$+ \sum_{\mathbf{Q}'} \mathrm{W}_{\mathbf{QQ}'}\left(\frac{N(\mathbf{Q}',t) - N(\mathbf{Q},t)}{2}\mathrm{I} + \mathbf{S}(\mathbf{Q}',t).\boldsymbol{\sigma} - \mathbf{S}(\mathbf{Q},t).\boldsymbol{\sigma}\right)$$

$$-\frac{1}{\tau}\left(\frac{N(\mathbf{Q},t)}{2}\mathrm{I} + \mathbf{S}(\mathbf{Q},t).\boldsymbol{\sigma}\right) + G$$

On equating the traceless matrices on both the sides of the above equation, we get:

$$\frac{d}{dt}\left(\mathbf{S}(\mathbf{Q},t).\boldsymbol{\sigma}\right) = \frac{i}{2}[\mathbf{S}(\mathbf{Q},t).\boldsymbol{\sigma}, \boldsymbol{\Omega}.\boldsymbol{\sigma}]$$

$$+ \sum_{\mathbf{Q}'} \mathrm{W}_{\mathbf{QQ}'}\left(\mathbf{S}(\mathbf{Q}',t).\boldsymbol{\sigma} - \mathbf{S}(\mathbf{Q},t).\boldsymbol{\sigma}\right) - \frac{1}{\tau}\left(\mathbf{S}(\mathbf{Q},t).\boldsymbol{\sigma}\right) + \mathbf{G}.\boldsymbol{\sigma}$$

where $\mathbf{G} = [G_x\ G_y\ G_z]$ is the generation rate vector.

By expanding the commutation bracket and using the vector identity $(\mathbf{S}.\boldsymbol{\sigma})(\boldsymbol{\Omega}.\boldsymbol{\sigma}) = (\mathbf{S}.\boldsymbol{\Omega})\mathrm{I} + i(\mathbf{S} \times \boldsymbol{\Omega}).\boldsymbol{\sigma}$, we get the final form of the MSS equation as:

$$\frac{d\mathbf{S}(\mathbf{Q})}{dt} = \boldsymbol{\Omega}(\mathbf{Q}) \times \mathbf{S}(\mathbf{Q}) + \sum_{\mathbf{Q}'} \mathrm{W}_{\mathbf{QQ}'}[\mathbf{S}(\mathbf{Q}') - \mathbf{S}(\mathbf{Q})] - \frac{1}{\tau}\mathbf{S}(\mathbf{Q}) + \mathbf{G}$$

Supplementary Note 2: Derivation that DOLP is given by $\langle S_x \rangle$

The state $|\psi\rangle$ in the basis states of two-orthogonal circular polarizations $|\sigma_{z+}\rangle, |\sigma_{z-}\rangle$ is expressed as:

$$|\psi\rangle = \cos\frac{\theta}{2}|\sigma_{z+}\rangle + \sin\frac{\theta}{2}e^{i\phi}|\sigma_{z-}\rangle$$

Thus, the degree of circular polarization (DOCP) of this single exciton qubit state can be obtained as:

$$\mathrm{DOCP} = \frac{\left(\cos^2\frac{\theta}{2} - \sin^2\frac{\theta}{2}\right)}{\left(\cos^2\frac{\theta}{2} + \sin^2\frac{\theta}{2}\right)} = \cos\theta = S_z$$

Similarly, in the basis states of the $|\sigma_{x+}\rangle, |\sigma_{x-}\rangle$ vectors that represent the two orthogonal linear polarization directions [vertical (V) and horizontal (H)], the state can also be represented as

$$|\psi\rangle = \frac{1}{\sqrt{2}}\left(\cos\frac{\theta}{2} + \sin\frac{\theta}{2}e^{i\phi}\right)|\sigma_{x+}\rangle + \frac{1}{\sqrt{2}}\left(\cos\frac{\theta}{2} - \sin\frac{\theta}{2}e^{i\phi}\right)|\sigma_{x-}\rangle$$

in which case, the degree of linear polarization (DOLP) can be calculated as:

$$\mathrm{DOLP} = \frac{\left(\left|\cos\frac{\theta}{2} + \sin\frac{\theta}{2}e^{i\phi}\right|^2 - \left|\cos\frac{\theta}{2} - \sin\frac{\theta}{2}e^{i\phi}\right|^2\right)}{\left(\left|\cos\frac{\theta}{2} + \sin\frac{\theta}{2}e^{i\phi}\right|^2 + \left|\cos\frac{\theta}{2} - \sin\frac{\theta}{2}e^{i\phi}\right|^2\right)} = \sin\theta\cos\phi = S_x$$

The overall steady state DOLP is then obtained as $\langle S_x\rangle$ averaged over the **Q** space.

Supplementary Note 3: DOLP is greater than DOCP in a 2D system

To show that the exciton DOLP is generally higher than its DOCP value in 2D semiconductors, we take a special case of no scattering scenario ($\mathrm{W}_{\mathbf{QQ}'} \to 0$). The steady-state form of the MSS equation is then given by:

$$\mathbf{G} = \frac{1}{\tau}\mathbf{S}(\mathbf{Q}) - \mathbf{\Omega}(\mathbf{Q}) \times \mathbf{S}(\mathbf{Q})$$

$\mathbf{\Omega} = [\Omega_{\parallel}\cos\phi,\ \Omega_{\parallel}\sin\phi, 0]$ in the absence of an external magnetic field, and ϕ is the azimuthal angle between **Q** and x axis.

The steady state value of S_z can be obtained by putting $G_x = 0, G_y = 0\ and\ G_z = G$ in the above equation as

$$S_z(\mathbf{Q}) = \frac{G\tau}{(1 + \tau^2\Omega_{\parallel}^2(\mathbf{Q}))}$$

Similarly, the steady state value of S_x can be obtained by putting $G_x = G, G_y = 0\ and\ G_z = 0$:

$$S_x(\mathbf{Q}) = \frac{G\tau\ (1 + \tau^2\Omega_{\parallel}^2(\mathbf{Q})\cos^2 2\phi)}{(1 + \tau^2\Omega_{\parallel}^2(\mathbf{Q}))}$$

After the initial generation along the $0^0, 180^0$ axis, the degree of linear polarization $S_x(\mathbf{Q})$ at those **Q** values in the limiting condition of $\mathrm{W}_{\mathbf{QQ}'} \to 0$ ($\phi \to 0^0, 180^0$) is $G\tau$. Hence, we can see that, in the case of no scattering, the pseudospin S_x is nearly independent of $\Omega_{\parallel}$, whereas

S_z suffers due to $\Omega_{\|}$. A physical explanation for this is that the linear polarization degrades because of one of the in-plane magnetic field components, but the out-of-plane circular polarization experiences the effect of both the in-plane components of the exchange magnetic field[1,2]. As a result, the DOLP is generally higher than the DOCP in these 2D exciton.

Supplementary Note 4: Simulation Details

4.1. Electronic and Excitonic band structure calculation

We use 2×2 **k.p** Hamiltonian to get the band dispersion of the lowest energy conduction band (CB) and highest energy valence band (VB) in the $\mathbf{K}, \mathbf{K}'$ valley in monolayer TMDs. The Hamiltonian in the atomic orbital basis states of $|d_{z^2}\rangle$ and $\frac{1}{\sqrt{2}}\left(|d_{x^2-y^2}\rangle + i\tau|d_{xy}\rangle\right)$, where τ $(=\pm 1)$ represents the valley index, is given by[3]:

$$\begin{bmatrix} E_g & at(\tau k_x - ik_y) \\ at(\tau k_x + ik_y) & 0 \end{bmatrix}$$

k_x, k_y are the wave vectors in the reciprocal space. The value of the lattice constant a, the hopping amplitude t, and the quasi-particle band gap E_g for calculating monolayer MoS_2 band structure are taken as 3.193 Å, 1.10 eV and 2.4 eV, respectively. The eigen energies (eigen functions) obtained after solving the above Hamiltonian is denoted as $E_{c,\mathbf{k}}(|c, \mathbf{k}\rangle)$, $E_{v,\mathbf{k}}(|v, \mathbf{k}\rangle)$ for the conduction and valence band, respectively.

In the basis of the combined CB ($|c, \mathbf{k} + \mathbf{Q}\rangle$) and VB ($|v, \mathbf{k}\rangle$) pair states, denoted together as $|vc\mathbf{kQ}\rangle$, where $\mathbf{Q}$ $(= \mathbf{k_e} + \mathbf{k_h})$ is the exciton center-of-mass momentum, the exciton band structure is calculated by solving the following Bethe-Salpeter equation[4]:

$$\langle vc\mathbf{kQ}|H|vc\mathbf{k}'\mathbf{Q}\rangle = \delta_{kk'}\left(E_{c,\mathbf{k}+\mathbf{Q}} - E_{v,\mathbf{k}}\right) - (D - X)(\mathbf{k}, \mathbf{k}', \mathbf{Q})$$

D, X are the direct and the exchange interaction terms. We neglect the contribution of exchange interaction at small $\mathbf{Q}$ within the light cone in the exciton band structure calculation. The direct coulombic interaction is given by:

$$D = \frac{1}{A} V_{k-k'} \langle c, \mathbf{k} + \mathbf{Q}|c, \mathbf{k}' + \mathbf{Q}\rangle \langle v, \mathbf{k}'|v, \mathbf{k}\rangle$$

Here A is the area of the two-dimensional crystal and $V_{k-k'}$ is the interaction potential given by[5]:

$$V_q = \frac{2\pi {q_0}^2 e^{-q\xi}}{q} \frac{1}{\epsilon(q)}$$

Here q_0 is the magnitude of the charge of an electron, $q = |\mathbf{k} - \mathbf{k}'|$, and ξ is a fitting parameter that reflects the extension of the wavefunction of the electron and the hole in the out-of-plane direction. The dielectric function $\epsilon(q)$ is given by

$$\epsilon(q) = \frac{(1 - p_b p_t e^{-2\eta q D})\kappa}{(1 - p_t e^{-\eta q D})(1 - p_b e^{-\eta q D})} + r_0 q e^{-q\xi}$$

For the case of identical dielectric environment on top and bottom, $p_b = p_t = P = (\epsilon_{ENV} - \kappa)/(\epsilon_{ENV} + \kappa)$. D is the thickness of the monolayer sheet, $\eta = \sqrt{\epsilon_{\parallel}/\epsilon_{\perp}}$ and $\kappa = \sqrt{\epsilon_{\parallel}\epsilon_{\perp}}$. $\epsilon_{\parallel}$ $(\epsilon_{\perp})$ and ϵ_{ENV} is the effective in-plane (out-of-plane) and environmental dielectric constant of the monolayer TMD. We use the high frequency dielectric constant value of $\epsilon_{\parallel} = 5.01$ and $\epsilon_{\perp} = 6.07$ in our calculations.

The fitting parameters for the HMH, GHMHG and the GMG stacks are summarized below:

	ϵ_{ENV}	r_0 (m)	ξ(Å)
HMH	4	12×10^{-8}	8.64
GHMHG	16	23.6×10^{-8}	20
GMG	20	32×10^{-8}	25.6

With these fitting parameters, we ensure that the calculated $A_{2s} - A_{1s}$ energy separation matches with the experimentally obtained value. The exciton energy spectrum for the first few states showing the A_{1s} exciton binding energy change in the three stacks is plotted in the main text (Figure 3d), and the full spectrum is shown below.

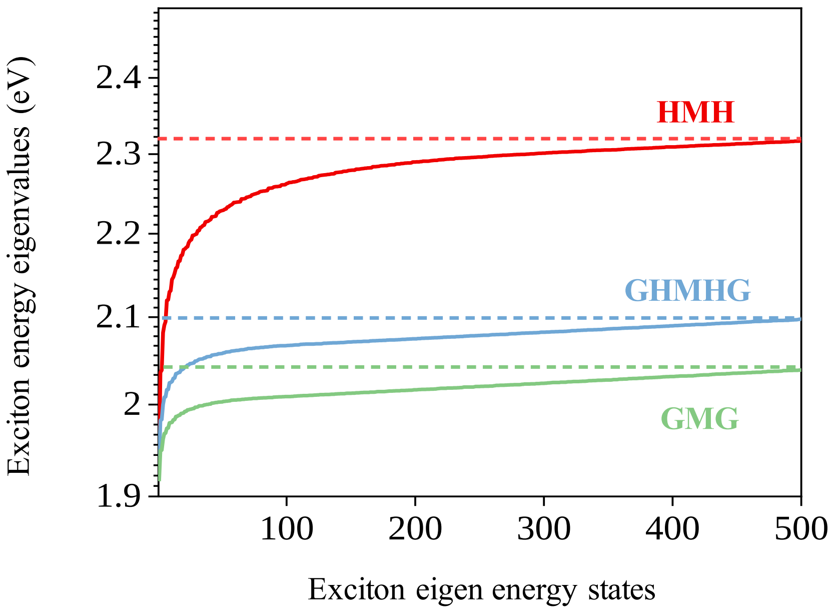

Figure S2: The calculated exciton eigen energy spectrum (solid lines) for the HMH (in red), GHMHG (in blue), and the GMG (in green) stack obtained after solving the Bethe-Salpeter equation. The dashed lines represent the respective continuum energy levels. The extracted A_{1s} exciton binding energy is 379 meV in the HMH stack. The binding energy reduces to 162.5 and 122 meV in the GHMHG and the GMG stack, respectively due to dielectric screening.

4.2. DOLP calculation

The DOLP for the three stacks is calculated using the steady-state form of the MSS equation given as:

$$\mathbf{G} = \frac{1}{\tau}\mathbf{S}(\mathbf{Q}) - \boldsymbol{\Omega}(\mathbf{Q}) \times \mathbf{S}(\mathbf{Q}) - \sum_{\mathbf{Q}'} \mathrm{W}_{\mathbf{QQ}'}[\mathbf{S}(\mathbf{Q}') - \mathbf{S}(\mathbf{Q})]$$

τ is the net exciton lifetime, $\mathbf{G}$ is the exciton generation rate, $\mathrm{W}_{\mathbf{QQ}'}$ is the exciton scattering rate within the light cone, and $\Omega_{\parallel}(\mathbf{Q})$ $(= 2|J_{\mathbf{Q}}^{LR}|/\hbar)$ is the precession frequency magnitude due to intervalley exchange induced magnetic field, where $J_{\mathbf{Q}}^{LR}$ is the long-range component of exchange, and is given by[6]:

$$J_{\mathbf{Q}}^{LR} = -\frac{|\sum_{\mathbf{k}} \psi(\mathbf{k})|^2}{A} \frac{a^2 t^2}{E_g^2} V(\mathbf{Q}) \, |\mathbf{Q}|^2 e^{-2i\phi}$$

ϕ is the azimuthal angle in the $\mathbf{Q}$-space. The screening modifies the exchange interaction through the change in the electron-hole wavefunction overlap $|\sum_{\mathbf{k}} \psi(\mathbf{k})|^2/A$, bandgap E_g and the Fourier potential $V(\mathbf{Q})$. In Figure 3e of the main text, the magnitude of the exchange interaction potential is plotted as a function of $|\mathbf{Q}|$ within the light cone. During the calculation, we fit the parameters such that the $A_{2s} - A_{1s}$ separation remains the same as that obtained experimentally. τ is the exciton lifetime. For $\mathrm{W}_{\mathbf{QQ}'}$, we take the exciton-impurity scattering within the light cone as the only scattering mechanism at 5 K, neglecting exciton-phonon scattering. For simplicity, to get the exciton-impurity scattering matrix element, we use a similar form of the perturbing potential expression given for electron-ionized impurity scattering. We use the generic screened coulomb potential form as[7]

$$\frac{{q_0}^2}{4\pi\epsilon_0\epsilon_s r}\, e^{-r/L_D}$$

ϵ_0 is the dielectric permittivity of vacuum and $L_D$is the Debye length. The matrix element for the above perturbing potential for an exciton scattering from $\mathbf{Q}$ to $\mathbf{Q}'$ state is given by

$$H_{\mathbf{Q}'\mathbf{Q}} = \frac{1}{A}\left(\frac{{q_0}^2}{4\pi\epsilon_0\epsilon_s r}\right)\int e^{-i\mathbf{Q}'.\mathbf{r}}\,\frac{e^{-r/L_D}}{r}\,e^{i\mathbf{Q}.\mathbf{r}}\,d^2\mathbf{r}$$

$$= \left(\frac{{q_0}^2}{4\pi\epsilon_0\epsilon_s A}\right)\iint e^{i(\mathbf{Q}-\mathbf{Q}').\mathbf{r}}\,e^{-r/L_D}\,dr\,d\phi$$

Putting $\mathbf{Q}' - \mathbf{Q} = \boldsymbol{\beta}$ (note that, $|\mathbf{Q}'| = |\mathbf{Q}|$ since Coulomb scattering is elastic in nature), we obtain

$$H_{\mathbf{Q}'\mathbf{Q}} = \left(\frac{{q_0}^2}{4\pi\epsilon_0\epsilon_s A}\right)\iint e^{-i\beta r\cos\phi}\,e^{-r/L_D}dr\,d\phi$$

The above expression after integration over r results in

$$H_{\mathbf{Q}'\mathbf{Q}} = \left(\frac{{q_0}^2 L_D}{4\pi\epsilon_0\epsilon_s A}\right)\left[\int\frac{1}{1+\beta^2L_D^2\cos^2\phi}\,d\phi - i\int\frac{\beta L_D\cos\phi}{1+\beta^2L_D^2\cos^2\phi}\,d\phi\right]$$

The result of the first integration is $2\pi/\sqrt{1+\beta^2L_D^2}$ and the second one is zero. The matrix element thus becomes

$$H_{\mathbf{Q}'\mathbf{Q}} = \frac{{q_0}^2}{2\epsilon_0\epsilon_s A\sqrt{\beta^2 + 1/L_D^2}}$$

We assume the limiting case of $L_D \to \infty$ and obtain the following expression for the scattering rate $W_{\mathbf{Q'Q}}$ as

$$W_{\mathbf{Q'Q}} \propto \left|H_{\mathbf{Q'Q}}\right|^2 = \left(\frac{{q_0}^2}{2\epsilon_0\epsilon_s A\,\beta}\right)^2$$

where $\beta = \frac{2Q}{\hbar} \sin \alpha/2$, α is the angle between the initial $(\mathbf{Q})$ and the final $(\mathbf{Q}')$ exciton state. The final form of the steady state MSS equation after plugging in the above form of scattering rate $W_{\mathbf{Q'Q}}$ as given in the main text is given as:

$$\mathbf{G} = \frac{1}{\tau}\mathbf{S}(\mathbf{Q}) - \boldsymbol{\Omega}(\mathbf{Q}) \times \mathbf{S}(\mathbf{Q}) - \sum_{\mathbf{Q}'} \frac{w}{Q^2 \sin^2\frac{\alpha}{2}}[\mathbf{S}(\mathbf{Q}') - \mathbf{S}(\mathbf{Q})]$$

where w is used as a fitting parameter. Finally, the DOLP $(= \langle S_x \rangle)$ is calculated by averaging the calculated pseudospin at the $\mathbf{Q}$-values within the light cone and is plotted in Figure 4 in the main text for the HMH and the GMG sample.

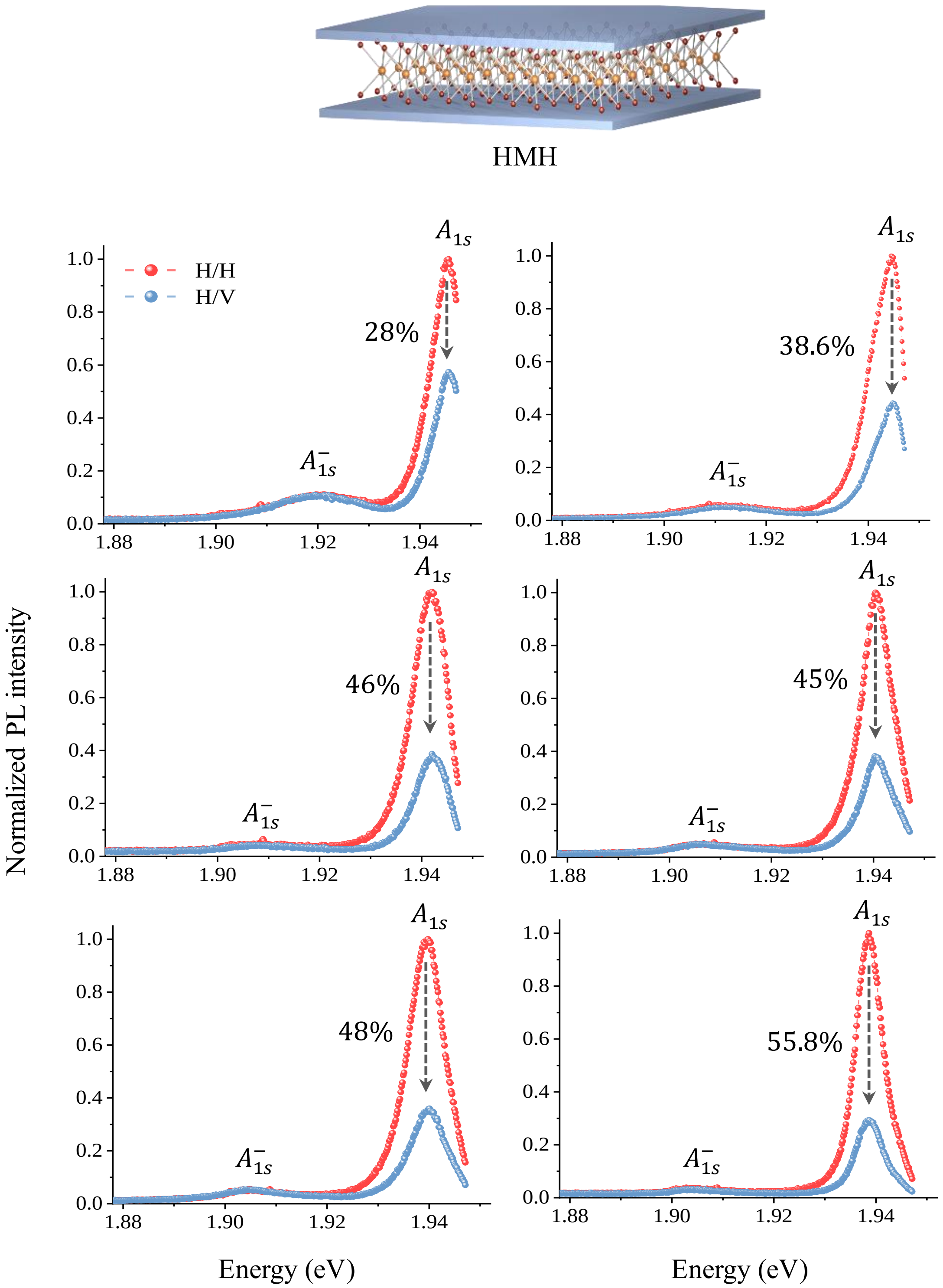

Figure S3: The co- (H/H, in red) and cross- (H/V, in blue) linearly polarized PL spectra obtained at different spots on the HMH stack in the increasing order of DOLP. The range of the DOLP values obtained lies between $26\ - 64$ %.

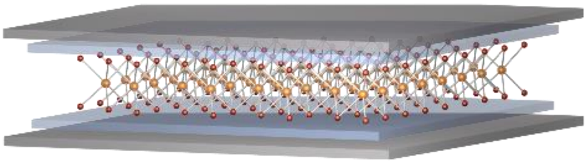

GHMHG

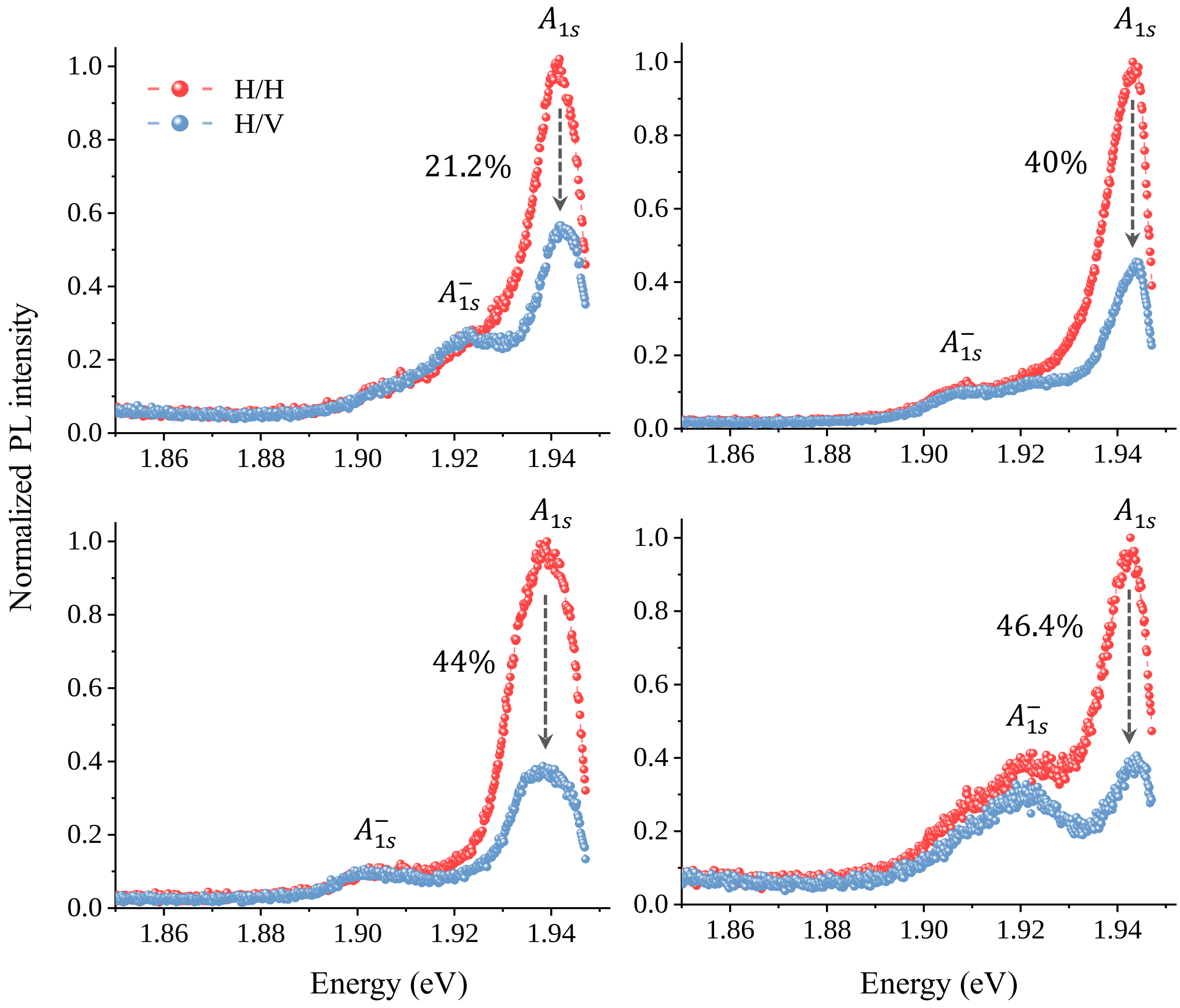

Figure S4: The co- (H/H, in red) and cross- (H/V, in blue) linearly polarized PL spectra obtained at different spots on the GHMHG stack. The DOLP values range within $21 - 46$ % in this stack.

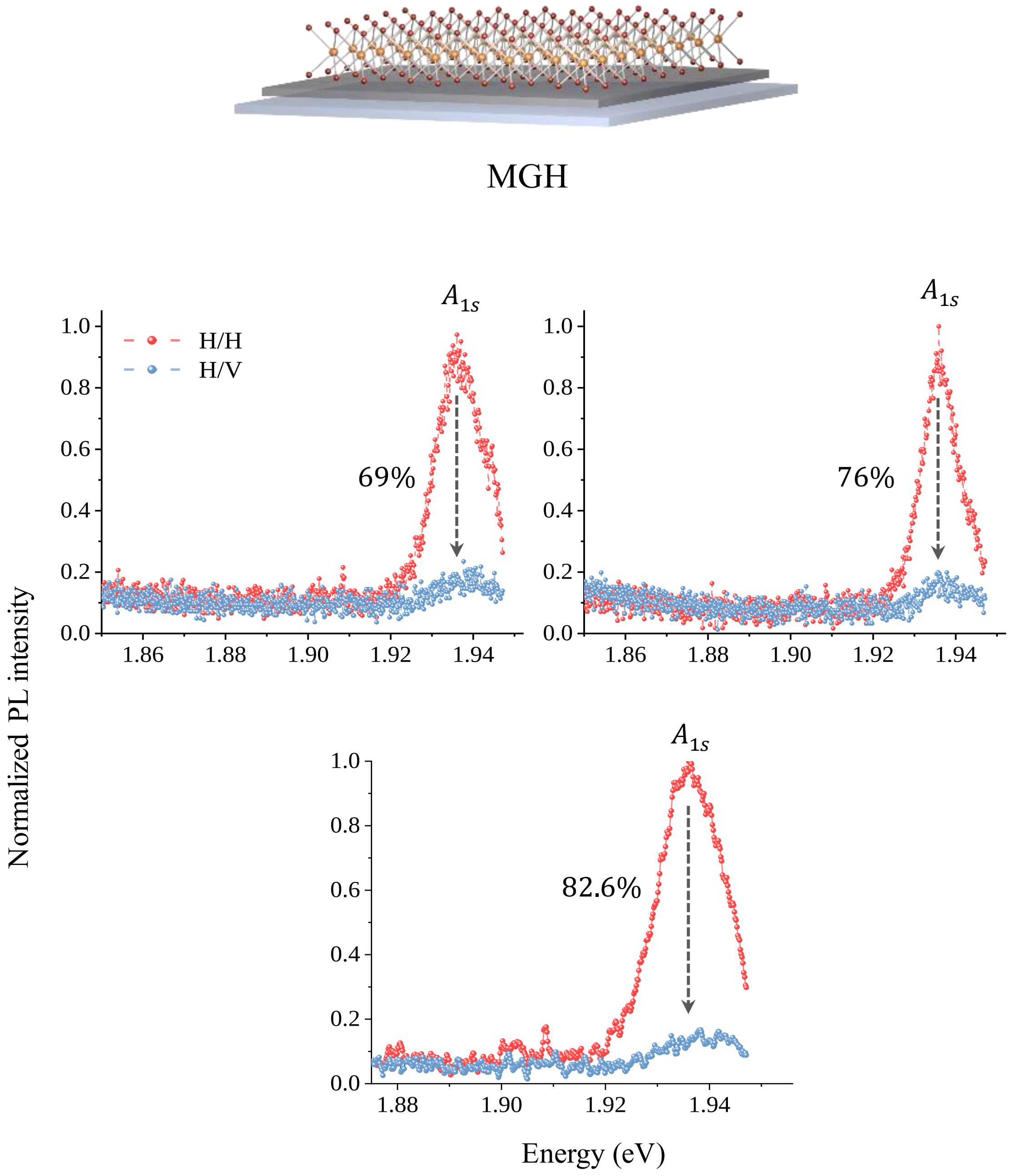

Figure S5: The co- (H/H, in red) and cross- (H/V, in blue) linearly polarized PL spectra obtained at different spots on the MGH stack. The DOLP values range within $69 - 82$ % in this stack.

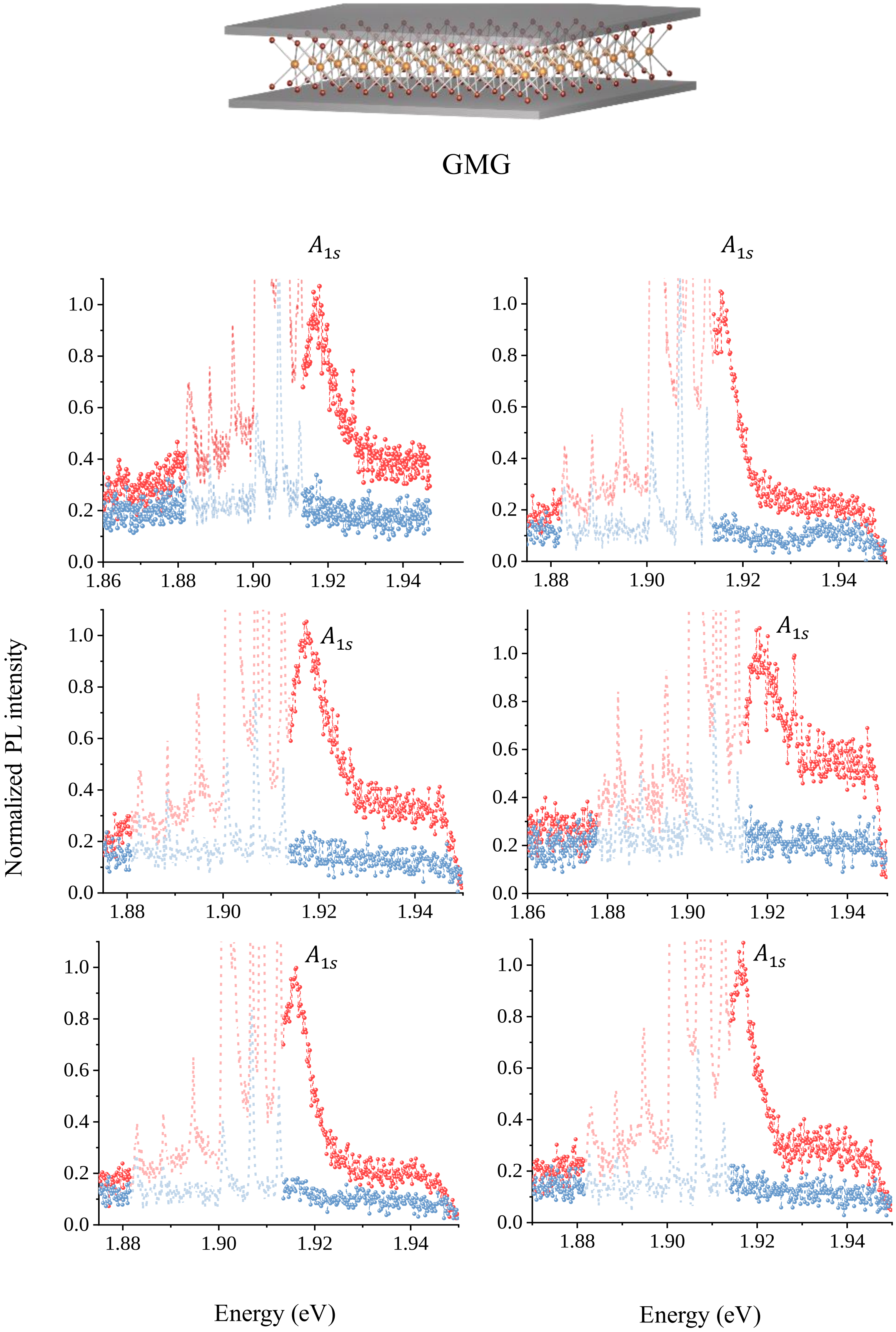
GMG
A_{1s}
Normalized PL intensity
Energy (eV)
Energy (eV)

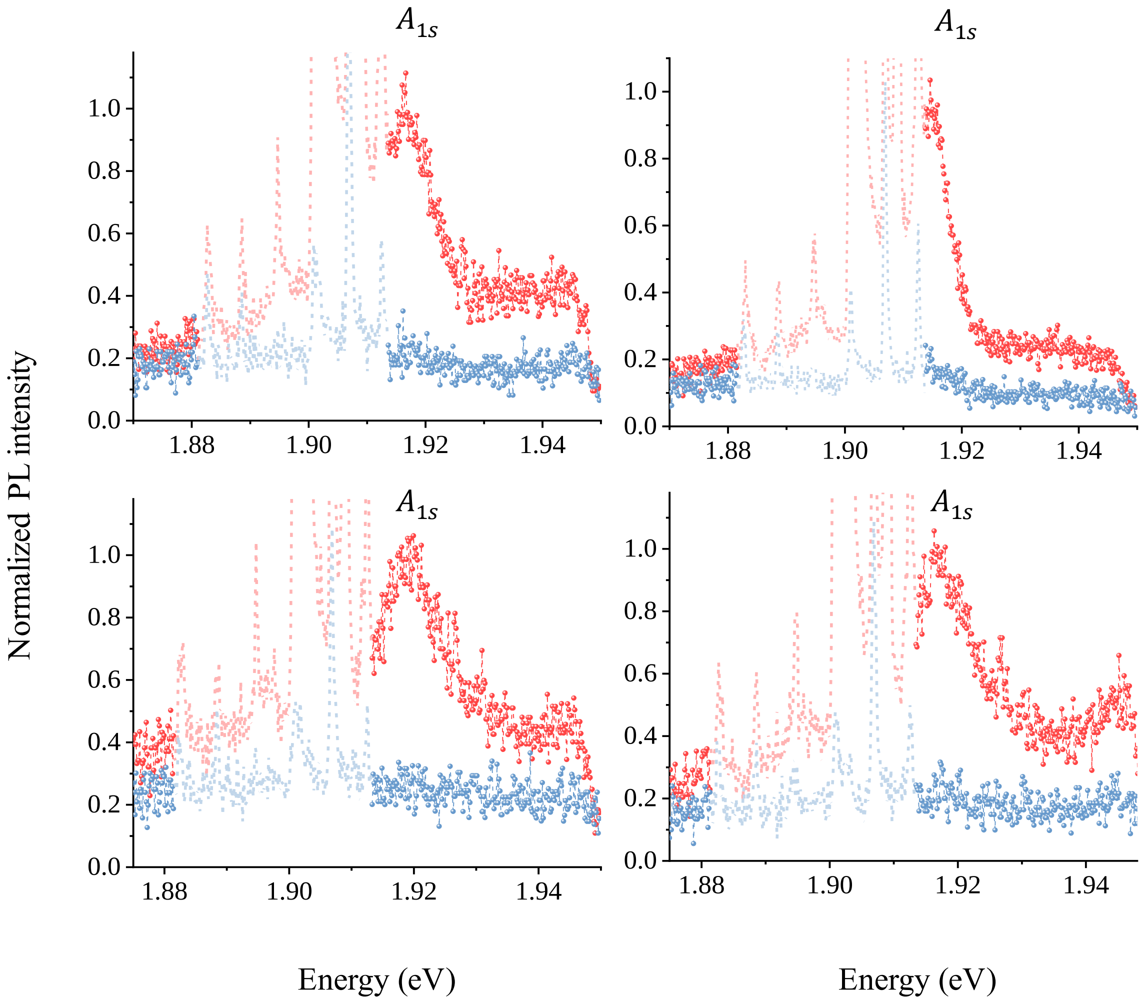

Figure S6: The co- (H/H, in red) and cross- (H/V, in blue) linearly polarized PL spectra taken at different spots on the GMG showing 100% DOLP. The peaks indicated by the dashed lines represent Raman peaks. The dashed lines are the strong Raman peaks due to dual resonance in the GMG stack.

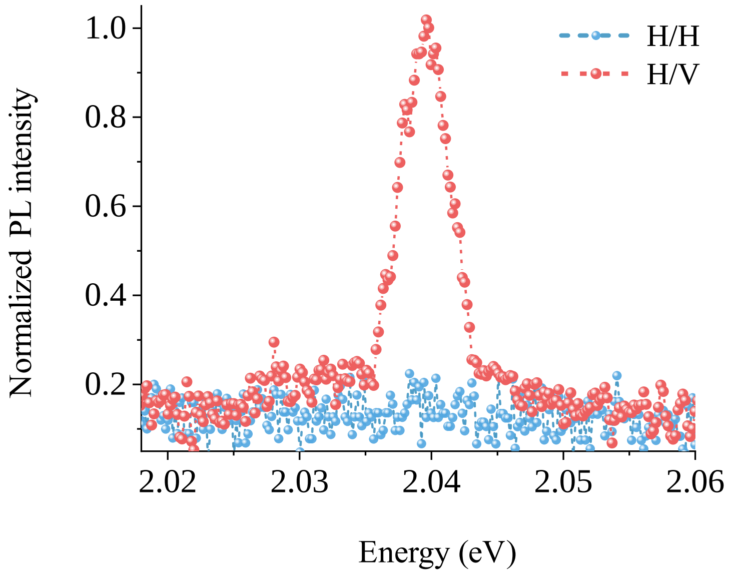

Figure S7a: The co- (H/H, in red) and cross- (H/V, in blue) linearly polarized PL spectra taken on the few-layer graphene encapsulated WS_2 (GWG) sample showing ~100% DOLP at 5 K. The excitation wavelength is 593 nm.

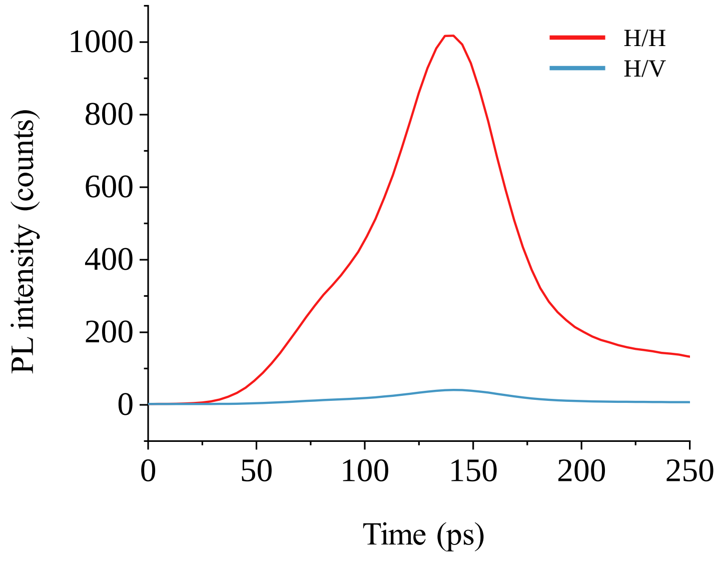

Figure S7b: Polarization Resolved TRPL measurement showing a peak DOLP of 97.6% in the GWG stack at 5 K obtained with near-resonant excitation at 593 nm.

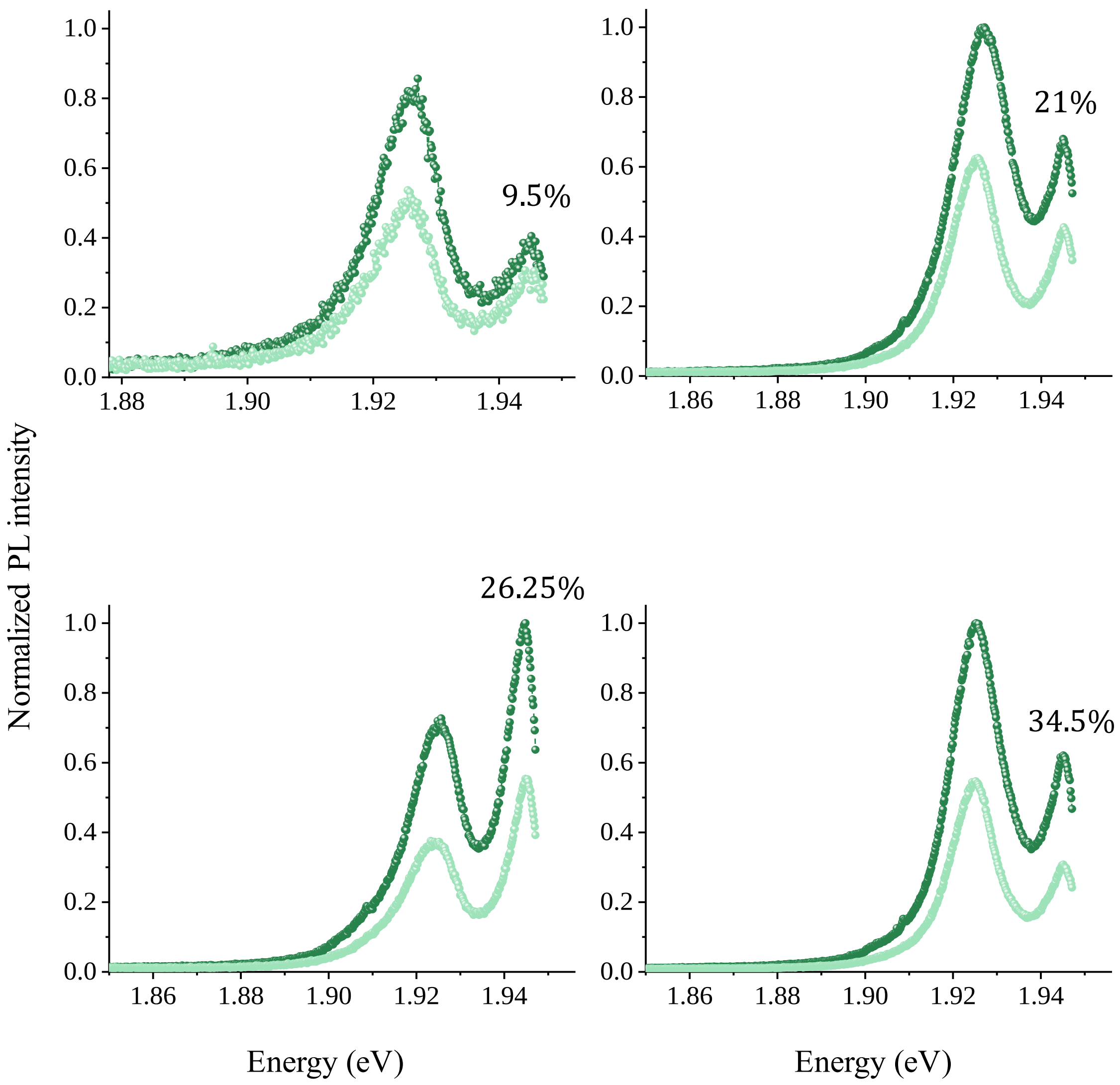

Figure S8: The co- ($\sigma+/\sigma+$, in dark green) and cross- ($\sigma+/\sigma-$, in light green) circularly polarized PL spectra obtained at different spots on the HMH stack.

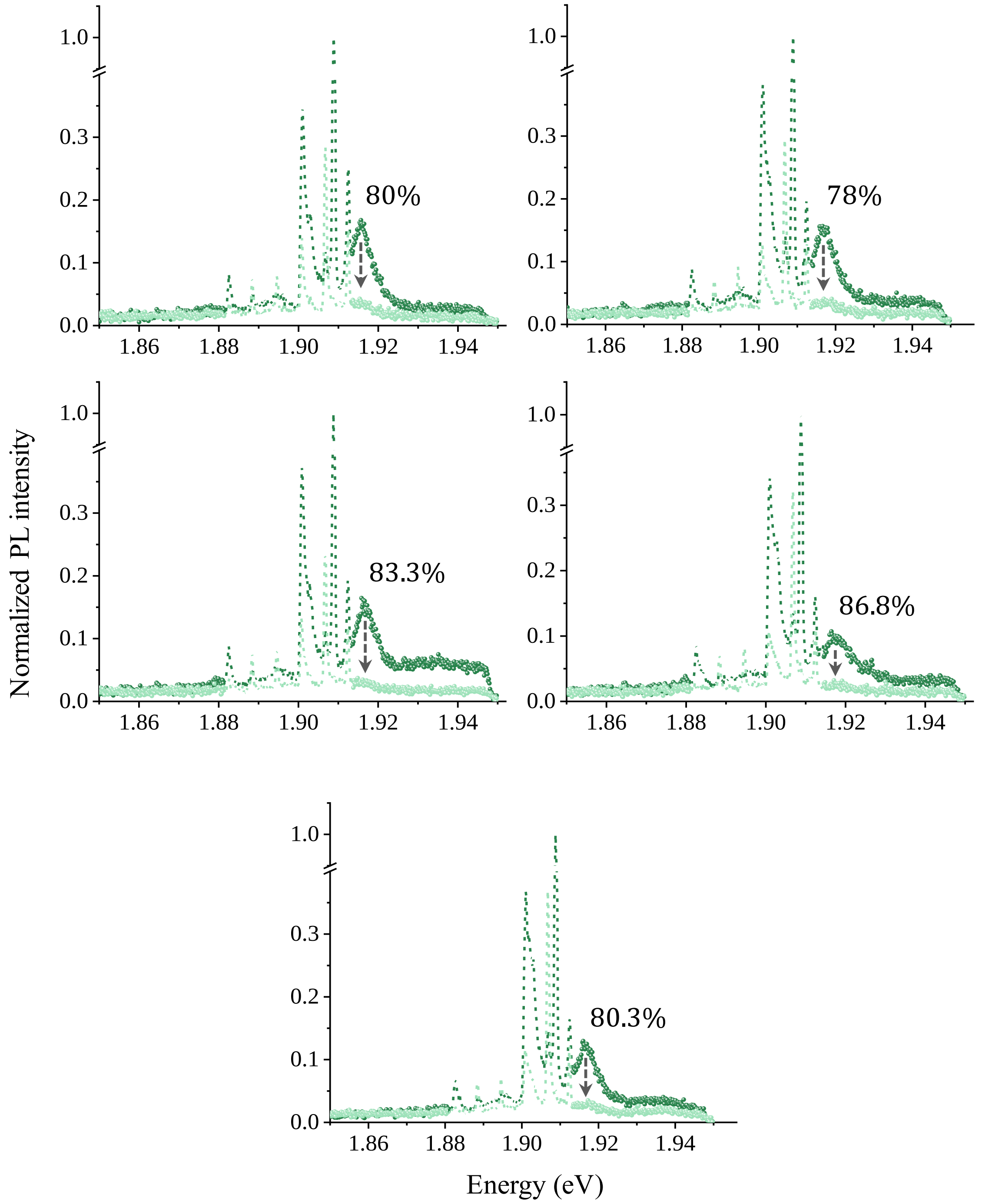

Figure S9: The co- ($\sigma+/\sigma+$, in dark green) and cross- ($\sigma+/\sigma-$, in light green) circularly polarized PL spectra obtained at different spots on the GMG stack. The peaks indicated by the dashed lines represent Raman peaks. The dashed lines are the strong Raman peaks due to dual resonance in the GMG stack.

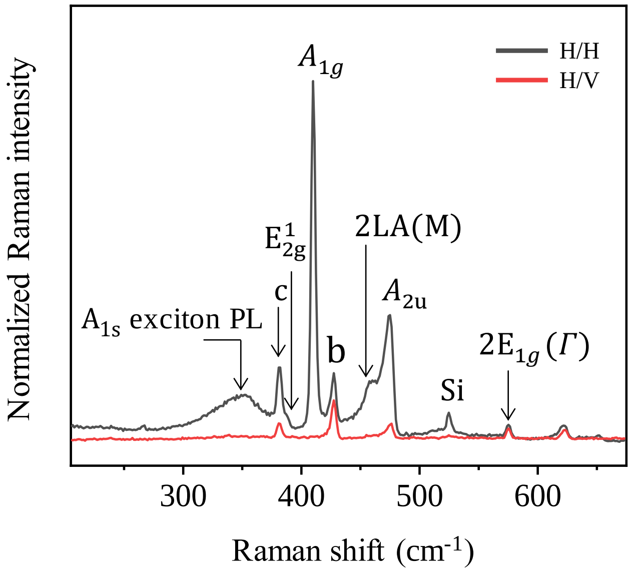

Figure S10: The linear polarization resolved Raman spectra of the GMG stack on 633 nm near-resonant excitation at 5 K. The nomenclature of the Raman peaks is adopted from Chakraborty et al.[8]

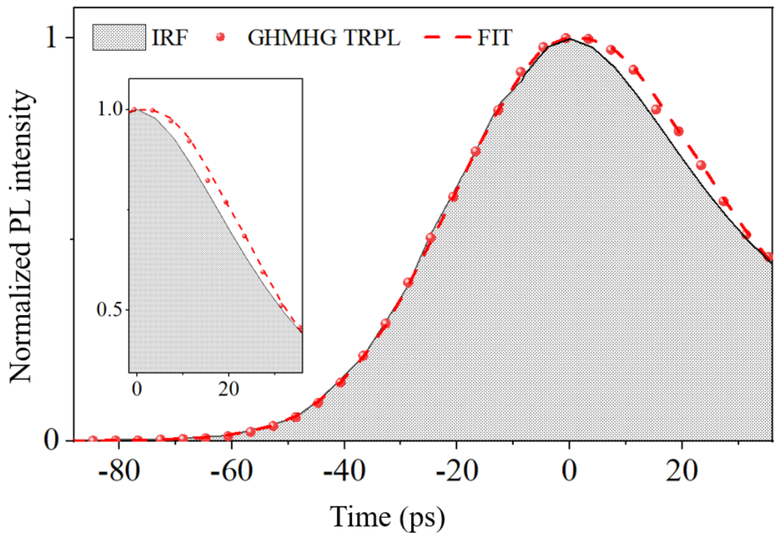

Figure S11: The time-resolved PL of the A_{1s} exciton in monolayer MoS_2 in the GHMHG stack taken at 5 K. The red symbols (red dashed trace) indicate the measured data (fitting). The instrument response function (IRF) is indicated by the grey shaded region. Inset: The zoomed-in view of the decay part in log scale.

We use the following procedure to deconvolute the IRF contribution and to extract the sample response. The response $[L(t)]$ of the system to an impulse excitation is given as follows:

$$L(t) = \sum_i A_i e^{-t/\tau_i}$$

Here A_i and τ_i represent the amplitude and time constant of the ith contributing species in the TRPL response, respectively. The above impulse response is convoluted with the experimentally obtained instrument response function (IRF) $[I(t)]$ (shaded region) iteratively, ensuring that the final convoluted output (dashed line) precisely matches the experimentally obtained TRPL data $[T(t)]$ (red symbols), i.e.,

$$T(t) = \int_0^t I(t')L(t-t')dt'$$

The number in the inset of the figure above represents the fastest component in the decay indicating the exciton lifetime at one of the spots.

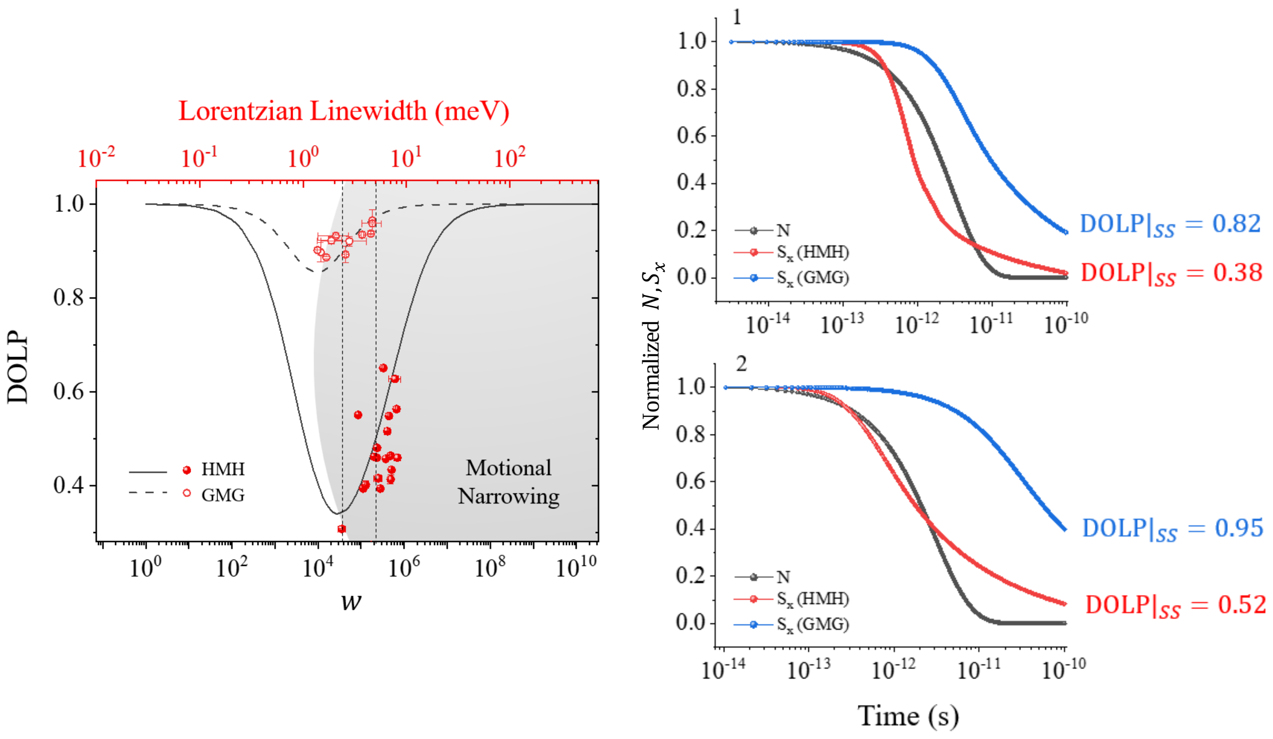

Figure S12: Left panel - Simulation results of the steady-state MSS equation (lines) and experimental (symbols) results comparing the exciton DOLP as a function of w (bottom axis) and Lorentzian linewidth (top axis) [Figure 4 in the main text]. The shaded region indicates motional narrowing. Right panel – Simulation results of the time-dependent MSS equation for the HMH (in red) and the GMG (in blue) stack. The top and the bottom plot corresponds to two different values of w, as marked by dashed vertical lines in the left panel. To verify the simulation results, we estimate the steady-state DOLP value using the following expression:

$$\mathrm{DOLP}|_{ss} = \frac{\sum_t \mathrm{N(t)}\, S_x(\mathrm{t})}{\sum_t \mathrm{N(t)}}$$

The steady-state DOLP value for the HMH and the GMG stack is also shown in the above plot and is in good agreement with the experimentally obtained DOLP values at that w. The time-dependent results of the MSS equation clearly show the giant enhancement of the valley coherence time in the GMG stack as compared to the HMH stack.

References

1. Maialle, M. Z., De Andrada E Silva, E. A. & Sham, L. J. Exciton spin dynamics in quantum wells. *Phys. Rev. B* **47**, 15776–15788 (1993).

2. Chen, S. Y. *et al.* Superior Valley Polarization and Coherence of 2s Excitons in Monolayer WSe2. *Phys. Rev. Lett.* **120**, 046402 (2018).

3. Xiao, D. et al. Coupled spin and valley physics in monolayers of MoS 2 and other group-VI dichalcogenides. *Phys. Rev. Lett.* **108.19**, 196802 (2012).

4. Wu, F., Qu, F. & Macdonald, A. H. Exciton band structure of monolayer MoS2. *Phys. Rev. B* **91**, 75310 (2015).

5. Van Tuan, D., Yang, M. & Dery, H. Coulomb interaction in monolayer transition-metal dichalcogenides. *Phys. Rev. B* **98**, 125308 (2018).

6. Yu, H. *et al*. Dirac cones and Dirac saddle points of bright excitons in monolayer transition metal dichalcogenides. *Nat. Commun.* **5**, 1–7 (2014).

7. Lundstrom, M. Carrier scattering. in *Fundamentals of Carrier Transport* 54–118 (Cambridge University Press, 2010). doi:10.1017/cbo9780511618611.005.

8. Chakraborty, B. *et al*. Layer-dependent resonant Raman scattering of a few layer MoS_2. *J. Raman Spectrosc.* **44**, 92–96 (2013).
